# The effects of electrical field spatial spread and some cognitive factors on speech-in-noise performance of individual cochlear implant users—A computer model study

**DOI:** 10.1371/journal.pone.0193842

**Published:** 2018-04-13

**Authors:** Tim Jürgens, Volker Hohmann, Andreas Büchner, Waldo Nogueira

**Affiliations:** 1 Medizinische Physik, Cluster of Excellence “Hearing4all” and Forschungszentrum Neurosensorik, Carl-von-Ossietzky Universität Oldenburg, Germany; 2 Medical University Hannover, Cluster of Excellence “Hearing4all”, Hannover, Germany; Universidad de Salamanca, SPAIN

## Abstract

The relation of the individual speech-in-noise performance differences in cochlear implant (CI) users to underlying physiological factors is currently poorly understood. This study approached this research question by a step-wise individualization of a computer model of speech intelligibility mimicking the details of CI signal processing and some details of the physiology present in CI users. Two factors, the electrical field spatial spread and internal noise (as a coarse model of the individual cognitive performance) were incorporated. Internal representations of speech-in-noise mixtures calculated by the model were classified using an automatic speech recognizer backend employing Hidden Markov Models with a Gaussian probability distribution. One-dimensional electric field spatial spread functions were inferred from electrical field imaging data of 14 CI users. Simplified assumptions of homogenously distributed auditory nerve fibers along the cochlear array and equal distance between electrode array and nerve tissue were assumed in the model. Internal noise, whose standard deviation was adjusted based on either anamnesis data, or text-reception-threshold data, or a combination thereof, was applied to the internal representations before classification. A systematic model evaluation showed that predicted speech-reception-thresholds (SRTs) in stationary noise improved (decreased) with decreasing internal noise standard deviation and with narrower electric field spatial spreads. The model version that was individualized to actual listeners using internal noise alone (containing average spatial spread) showed significant correlations to measured SRTs, reflecting the high correlation of the text-reception threshold data with SRTs. However, neither individualization to spatial spread functions alone, nor a combined individualization based on spatial spread functions and internal noise standard deviation did produce significant correlations with measured SRTs.

## Introduction

Cochlear implant (CI) users experience greater difficulty than normal-hearing (NH) listeners to understand speech when background noise is present. In addition to this general problem, speech-in-noise performance also varies considerably across CI users (e.g., [1]). Some CI users show speech understanding that is comparable to that of moderately hearing impaired listeners, whereas in others a speech reception threshold (SRT) in background noise cannot be specified, because 50% speech understanding cannot be reached even in quiet.

Many individual factors of CI users may influence their speech-in-noise performance. One factor that is widely discussed in the literature is the limited spectral resolution available to the CI user compared to NH listeners. Spectral resolution in CI users can be assessed in different ways. Objective (physical) measures include electrical field imaging (EFI, [2],[3],[4]) and electric compound action potentials (ECAPs, [5]), which offer electrode-specific and thus frequency-specific measures of the electrical field spatial spread in the cochlea. Subjective (perceptual) measures include place pitch discrimination [6], spatial tuning curves [7], and electrode discrimination [8]. These subjective measures also characterize spectral resolution frequency-specifically, whereas other subjective measures such as spectral ripple discrimination or detection [9] and spectral modulation thresholds [10] usually employ broadband stimuli with variable spectral contrast, which are more similar to speech.

Direct strong relations between spectral resolution and speech intelligibility in these studies have so far remained elusive. There are to our knowledge currently no links investigated between speech intelligibility and spatial spread assessed using EFI. Spatial spread assessed using ECAPs was not found to correlate significantly to speech-in-noise performance [11]. Subjective, frequency-specific measures show modest correlation to speech performance, such as for tuning curves inferred from gap detection [6], pitch ranking [12], or electrode discrimination [8], but other studies also show no correlations to speech performance (e.g., [13]). Correlations between speech performance to subjective spectral resolution measures with broadband stimuli show mixed results with some studies claiming strong correlations using, e.g., spectral modulation thresholds [10], but also studies which did not find such correlations [9], [14]. Possible reasons for these mixed results may be other individual factors involved in determining speech-in-noise performance, which limit the predictive power of the single factor spectral resolution.

Individual factors independent of spectral resolution that influence speech-in-noise performance are numerable. The most important investigated so far are age, duration of deafness, duration of hearing impairment, etiology [15], hearing aid usage [16], socioeconomic status, and a general cognitive ‘ability’, which can be measured using cognitive tests (e.g., [17]). The predictive power of these factors for speech performance either alone or combined is, however, relatively low, explaining less than typically 25% of the variance in speech tasks (e.g., [16]).

Computer model studies not involving human subjects allow systematic investigations of individual factors on the predicted speech in-noise-performance. Without comparison to actual CI users, however, these studies remain theoretic predictions. The human subject in these studies is replaced by a pattern recognizer that labels the processed acoustic signals (restricted by the factors investigated) according to its training and thus “recognizes” the speech items. The recognizer can either work with restricted training, for example in the form of a “frozen speech approach”, which means that exactly the same speech recording (and only one recording per item) is used for training and testing [18], [19], or with statistical speech models based on several recordings per speech item [20], [21], [22]. Fredelake and Hohmann [19] showed that wider electric field spatial spread functions that are uniform across electrodes resulted in higher SRTs and thus poorer speech-in-noise performance using restricted training. A similar trend was observed in their study when the cognitive ability was modelled by adjusting internal noise applied on the speech features. Stadler and Leijon [23] showed with a statistical speech recognition backend that an incorporation of a measure of spectral resolution has some predictive power for individually modelled SRTs. However, their work also shows how difficult it is to estimate spatial spread reliably and that such a reliable estimation is crucial for SRT-predictions, with large intra-individual differences across test-retest.

The current study aims at systematically analyzing the separate and combined effect of electrical field spatial spread and internal noise standard deviation on predicted speech-in-noise performance in a computer model for electric stimulation of the auditory system in combination with a statistical model of speech, by employing an automatic speech recognition system. Furthermore, it is investigated if an incorporation of one or the other factor, as newly collected in a group of individual CI users using Cochlear devices, improves the goodness of prediction of individual CI users’ speech-in-noise performance. Such a computer model approach allows to go beyond linear contributions of each of these factors to speech-in-noise performance, because both factors electrical field spatial spread and internal noise will nonlinearly interact within the model.

The manuscript is organized as follows: After a systematic evaluation about the effect of electrical field spatial spread and internal noise standard deviation in isolation on SRTs predicted by a physiologically-inspired computer model, the measurement data of individual CI users is investigated in terms of predictive power for measured SRTs using linear tools, such as correlation coefficients and a generalized linear model. The physiologically-inspired computer model is then individualized systematically to different degrees, based on measurement data: individualization based on spatial spread alone, internal noise alone, and combined individualizations are realized. Predicted and measured SRTs are compared, and the goodness of prediction is quantified.

## Methods

### Model structure

#### Model front end

This study uses the model front end of Fredelake and Hohmann [19], which is based on the dissertation of Hamacher [24]. The model is used here essentially as previously reported in [19], therefore, the model description will be kept brief.

**Fig 1:**
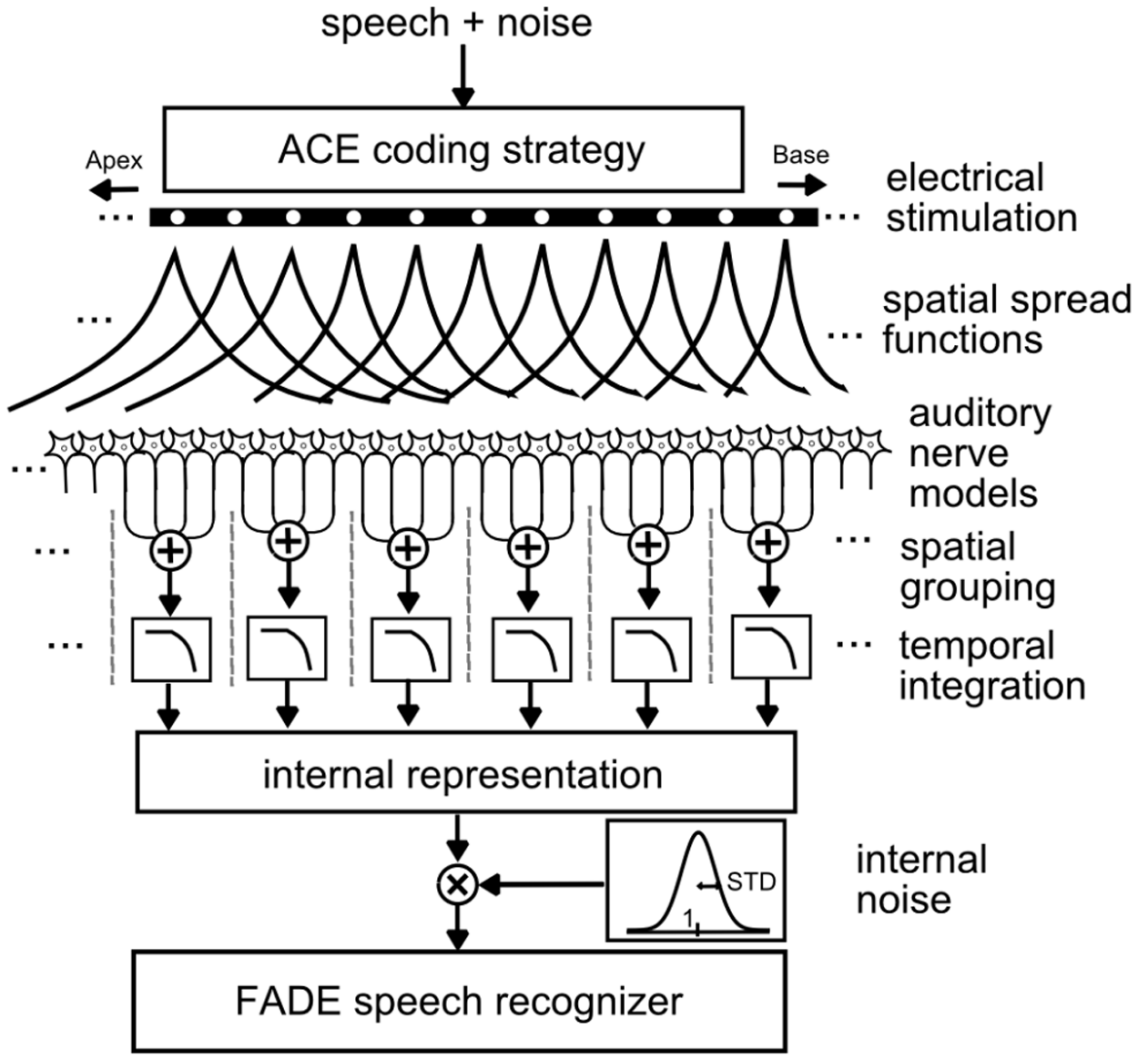
Sketch of the physiologically-inspired computer model used for the speech intelligibility predictions. The FADE speech recognizer serves as backend, whereas the other blocks up to “internal representation” serve as the model front-end. “Internal noise” is multiplied independently on each place-time bin of the internal representation prior to entering the FADE speech recognizer.

A sketch of the model structure is shown in Fig 1. The speech and noise mixture (at a given SNR) is first processed by the advanced combinational encoder (ACE) CI speech coding strategy (cf., [25]) giving an electrical pulse stimulation pattern on 22 electrodes. In agreement with [19], the electrodes were positioned centrally within a 35mm long, 1-dimensional cochlea. Subsequent to the electric stimuli, a spatial spread function on each of the 22 electrodes is used to simulate the transfer of the electric pulse onto each one of the auditory nerves, which were equally distributed along the entire length of the cochlea. In [19] and in experiment 1 of the current study, each spatial spread function is an idealized symmetrical double-sided exponential function with width λ (i.e., the distance from the center of the double-sided exponential to 1/e of the maximum amplitude) in millimeters. However, these spatial spread functions can also be individualized according to spatial spread functions measured in actual CI listeners. This spatial spread function serves as one of the major factors investigated in this study on speech-in-noise performance. The auditory nerves (AN) are modeled as leaky integrate-and-fire neurons with stochastically variable absolute and relative refractory times, latency and jitter, as well as a neuronal membrane noise. In the current study, 1000 AN cells were modelled. Afterwards, non-overlapping groups of adjacent auditory nerve cells are formed each associated with the electrode closest to the group. The spatial limits of each group are defined as the arithmetic midpoints between the position of the associated electrode and the positions of its left and right neighbors. Beyond the most basal and apical electrodes this grouping procedure is applied with a constant group width of 0.75 mm [19]. Spike trains within the groups are temporally integrated including a forward masking model. This results in an “internal representation” (IR), a spectrogram-like matrix of 46 rows, and columns at a frame update rate of 500 Hz. The excitation in each IR (amplitude of each time-frequency element) typically ranges between 0 and 50, in agreement with IRs shown in [19] (their Fig 5). Each element of the IR was multiplied with Gaussian noise (with a mean of 1 and a variable standard deviation, typically between 0.025 and 0.3), which is termed “internal noise”. This internal noise limits the predicted speech-in-noise performance and is used as the second major factor whose effect on individual and systematic SRTs is investigated in the current study.

#### Model backend

The Framework for auditory discrimination experiments (FADE) was used as speech pattern recognizer that provides a good generalization about the trained speech in the sense that it uses a statistical model generated from several speech utterances for a given word. The same framework was also used in combination with the electric model of Fredelake and Hohmann [19] in [26]. The details of this approach are given in [21] and will be briefly described here: 120 sentences of the Oldenburg sentence test mixed with stationary OLnoise, each at -12 dB SNR to 21 dB SNR in 3 dB steps were processed by the model front end resulting in whole-sentence IRs. This procedure was repeated 8 times for each SNR with different temporal passages of the noise, where 7 of these repetitions served as training and 1 as test material. Whole-word models with 6 states in a standard Hidden-Markov-Model (HMM) based on the Hidden-Markov-Model Toolkit (HTK, [27]) were trained using a Gaussian mixture model consisting of only one Gaussian distribution (with parameters mean and standard deviation). These models were used for the recognition of 600 presented words (contained within 120 sentences of 5 words each). Note that this approach does not receive separate words, but processes the entire sentence. The FADE framework automatically looks for word boundaries, because the HTK grammar was restricted to containing five subsequent words framed by a start silence model and a stop silence model. All combinations of training and testing SNRs were calculated resulting in combinations with low scores (at low SNRs) and high scores (with both training and testing having high SNRs at the same time), showing iso-score lines across different combinations. An interpolation between the two lowest testing SNRs along the 50%-iso-scoreline was then chosen as the predicted SRT. The motivation for this procedure was that also humans have acquired their speech discrimination and identification ability at a variety of different SNRs and should be able to make use of the “best-matching” training SNR to base their decision (in order to get best possible performance).

### Experiment 1: Systematic model evaluation

The aim of Experiment 1 was to systematically investigate the effect of spatial spread and internal noise on model-predicted SRTs. Therefore, SRTs were predicted as a function of different electrical field spatial spreads with a constant internal noise standard deviation σ_int_ = 0.19. This σ_int_ was chosen as the average strength used also in the individualization experiment 4 (see below). Furthermore, SRTs were predicted as a function of σ_int_ with constant electrical field spatial spread λ equal to 9 mm. The same spatial spread function for all electrodes was chosen within a given spatial spread (in mm), giving a homogenous array, for simplicity.

### Experiment 2: Linear models of measurement results from individual CI listeners

#### Participants

14 CI users aged between 34 and 85 years (median 64.5 years) participated in this study at the German Hearing Center of the Medical University Hanover. All participants were using Cochlear devices equipped with the ACE sound coding strategy and had at least 1 year of experience with their own CI. Therefore, the tested group of listeners was controlled for having the same device and signal processing strategy. For bilateral CI users only the side obtaining the best speech performance was tested. If a CI was worn on the other side, it was switched off during the measurements. Demographic information about the participants is shown in Table 1. The study protocol was approved by the institutional medical ethics committee of the Medical University of Hanover. All CI users gave their informed written consent to participate in the study.

**Table 1:** Demographic information about the participants of this study.

ID	Age	CI side investigated	Duration modest HL (years)	Duration profound HL (years)	Hearing aid usage before implantation	Years of experience with CI	Etiology
008	78	right	8	26	both sides	3	unknown
012	65	left	59	3	both sides	1	chronic otitis media
030	64	right	5	3	one side	3	sudden
031	47	right	37	<1	both sides	9	chronic otitis media
032	34	left	-	1	---	3	temporal bone fracture
041	85	left	15	19	both sides	1	sudden
044	44	left	33	<1	both sides	5	sudden
046	68	right	3	9	both sides	5	genetic
048	56	right	8	3	---	2	mixture of several causes
050	77	left	24	<1	both sides	3	sudden
060	78	right	---	42	one side	1	otosclerosis
061	38	left	22	9	both sides	1	genetic
062	38	right	26	9	both sides	2	since birth
063	68	right	10	33	one side	2	Menière’s disease

#### Electrical field measurements

The electrical potential distribution in the perilymph was measured using the Nucleus Interface Communicator (NIC; Cochlear Corp., Sydney, Australia) to stimulate and record from the electrodes of each CI user. It is known that the potential distribution depends on individual factors such as the geometry of the cochlea and the electrode positions. Each electrode was stimulated in monopolar mode using biphasic pulses with amplitude 106.50 μA, a phase width of 25 μs, and an inter-phase gap of 8 μs. The voltage was recorded on the same and on all the other electrodes, and normalized by the current amplitude of the stimulating biphasic pulses, resulting in an intra-cochlear potential map. Note that the physical unit of this normalized voltage is given in Ω. More details about the measurement procedure can be found in [28].

#### Text-reception-threshold test

An adjusted version of the Text-reception threshold (TRT) test [29], in detail described in [17], was used to assess the performance of the listener in visually combining fragments of words to a full sentence. This test displays sentences of the Oldenburg sentence test (e.g., “Peter kauft drei nasse Schuhe”, engl. “Peter buys three wet shoes”, [30]) on a computer screen and masks them with random bars, mimicking the masking effect of a fluctuating noise with speech-like modulation. The random bars masker was chosen, because this masker has shown highest correlations to SRTs in stationary noise (out of three tested masking patterns, [17]). The participant is asked to repeat the words that he/she can read. The percentage of sentence coverage with bars is adaptively adjusted during a measurement run (consisting of 20 sentences) until 50% of the words are correctly repeated. This coverage serves then as a non-audiological estimate about the ability of the participant to combine word fragments. Before the actual measurement data collection, two familiarization runs of 20 displayed sentences each were finished by each participant.

#### Anamnesis assessment

The participant’s anamnesis was assessed using a questionnaire, following procedures described in [16] and [15]. Age, year of first notice of the hearing loss, start of profound hearing loss (defined by inability to use the telephone), usage of hearing aids during the phase of profound hearing loss, year of implantation, and self-reported etiology were assessed on this questionnaire.

#### Speech intelligibility measurements

Speech intelligibility in noise was assessed using the Oldenburg sentence test (Wagener et al., 1999) adaptively, aiming at the SNR corresponding to 50% speech intelligibility (defined as SRT). Stationary, speech-shaped noise (OLnoise) and speech were presented using a frontal loudspeaker at 1 m distance to their own speech processor. The presentation level was set at 60 dB SPL (A). Two test lists were conducted in advance to the actual measurement to familiarize the CI user to the test.

### Extraction of parameters for model individualization

#### Electrical field spatial spread

The spatial spread of the electrical field in the perilymph was estimated by fitting single-sided exponential functions to each side of the off-diagonal elements of the intracochlear potential map, allowing a vertical offset to be present (i.e., exponential functions were not forced to approximate 0 for abscissa positions towards ± infinity). Separate offsets were chosen for the apical and basal ends of the curves, which allowed much better fits to the normalized voltage data than without. Within this manuscript the recordings at the stimulating electrode are disregarded, as these values are dominated by the electrode-tissue impedance and not by the anatomy [3]. A linear interpolation was done in the region of ±0.75 mm around the stimulating electrode and an extrapolation was done to regions of the cochlea not covered by the electrode array. The linear interpolation was done in contrast to [3] (who extended the exponential fits towards the stimulating electrode), because the steepness of some of the single-sided exponential functions would have resulted in extraordinary peaky spatial spread functions that would have dominated the signal transmission in the CI model. The procedure resulted in 22 spatial spread functions per CI user—one for each electrode.

**Fig 2:**
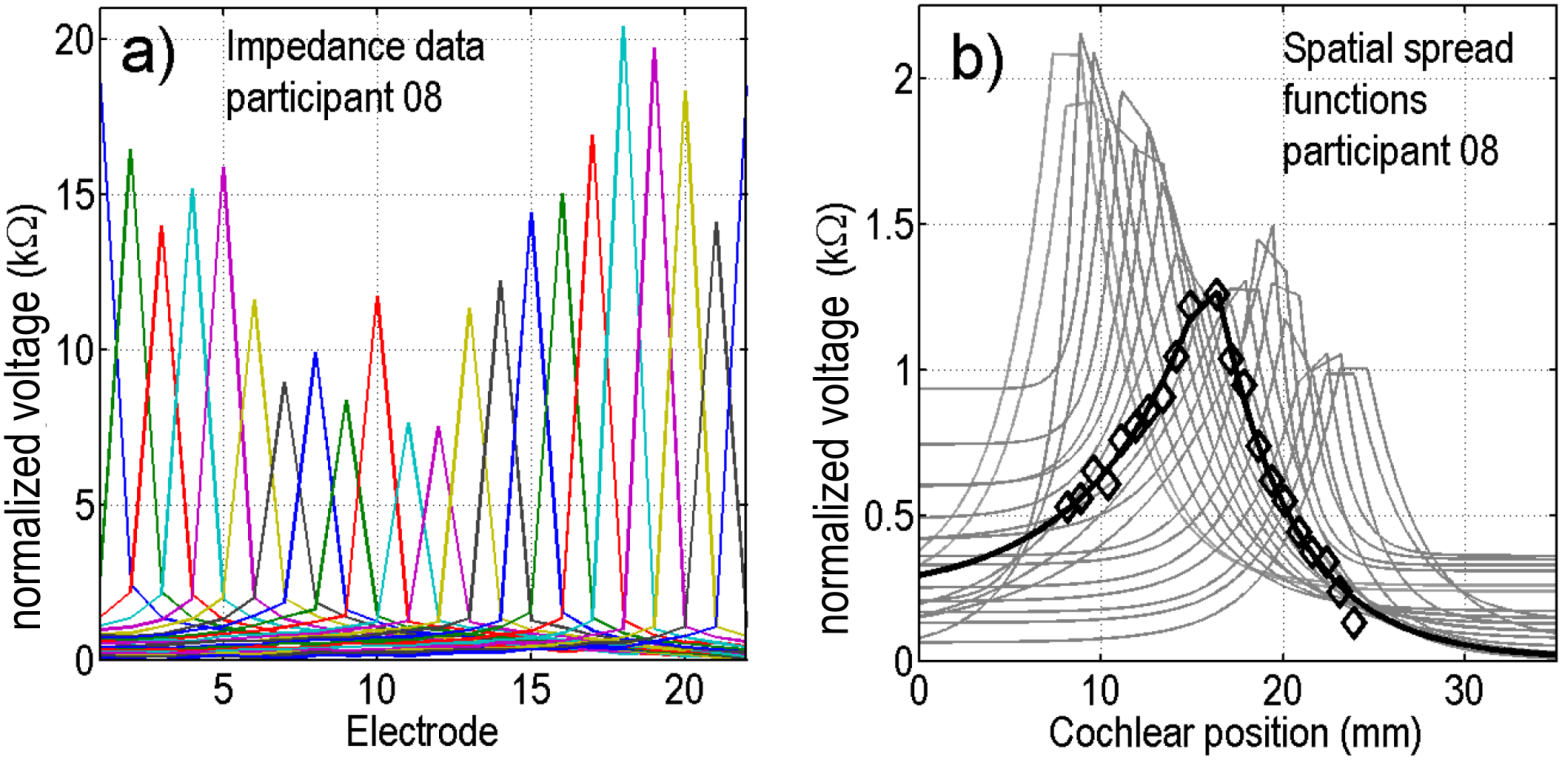
Exemplary spatial spread functions fitted to normalized voltage data of participant 08 for all 22 electrodes (gray lines). In addition, the raw normalized voltage data when stimulating at electrode 11 and measuring at all electrodes except for electrode 11 is plotted (black line and diamonds). Note that the raw normalized voltage values for the stimulating electrodes were omitted from the plots and fits, because they mainly reflect tissue impedance.

Fig 2 shows spatial spread functions (gray continuous lines) that were fitted to measured raw normalized voltage data for participant 08 as a typical example. For electrode 11 both the fitted spatial spread function (black continuous curve) and the 21 raw normalized voltage data points (black diamonds) are shown. The fit closely matches the measured data in the region of the cochlea covered by the electrodes. The spatial spread functions across the electrodes (gray lines) exhibit large differences in this participant.

**Fig 3:**
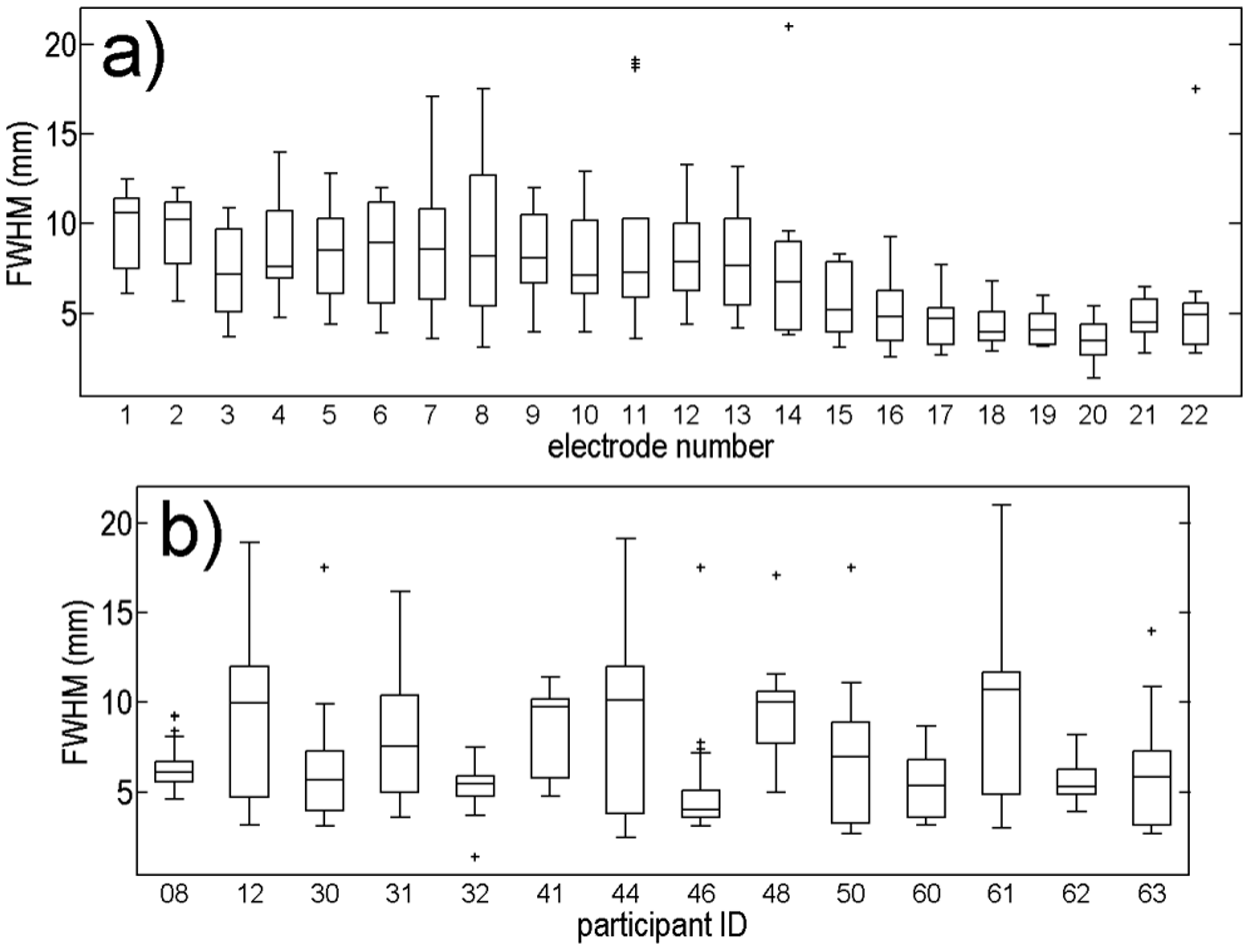
FWHM of fitted electric field spatial spread functions from normalized voltage data across electrodes (panel a, electrode 1 is most apical electrode) and across participants (panel b). Box plots denote median, 25% and 75% quartiles.

To quantify the width of each spatial spread function, full-width-half-maximum (FWHM) values were extracted from each fitted double-exponential curve as the full width halfway between the maximum and 0kΩ. Fig 3 shows the FWHM values of each fitted spatial spread function for each electrode (a) and each participant (b). FWHMs are highly variable across electrodes and across participants. There is a tendency to wider spatial spreads for low electrode numbers (more apical electrodes with a median of 10.3 mm for electrode 1) compared to narrower spatial spreads for high electrode numbers (more basal electrodes with a median of 5.0 mm for electrode 22). FWHMs of spatial spreads averaged across all electrodes are between 5.1 mm for participant 46 and 9.8 mm for participant 61.

#### Internal noise modelling

Internal noise standard deviation σ_int_ is adjusted in the current study using two different factors, which are the patient anamnesis and the cognitive performance of the patient. The phenomenological model of [16] and [15] was used to calculate the “auditory performance” (AP) from the factors assessed in the anamnesis questionnaire, which is a number (in %) that quantifies the expected detriment in speech recognition performance from the individual anamnesis data according to this phenomenological model. This factor may be interpreted as the deprivation of the auditory system preceding the implantation, which depends on duration of moderate and severe/profound hearing impairment, usage of hearing aids, age at implantation and etiology. In detail, the AP is calculated using Eq (1).

AP=Dur(mHL)·(−0.23%/y)+Dur(sHL)·Δs+B1+B2. (1)

**Fig 4:**
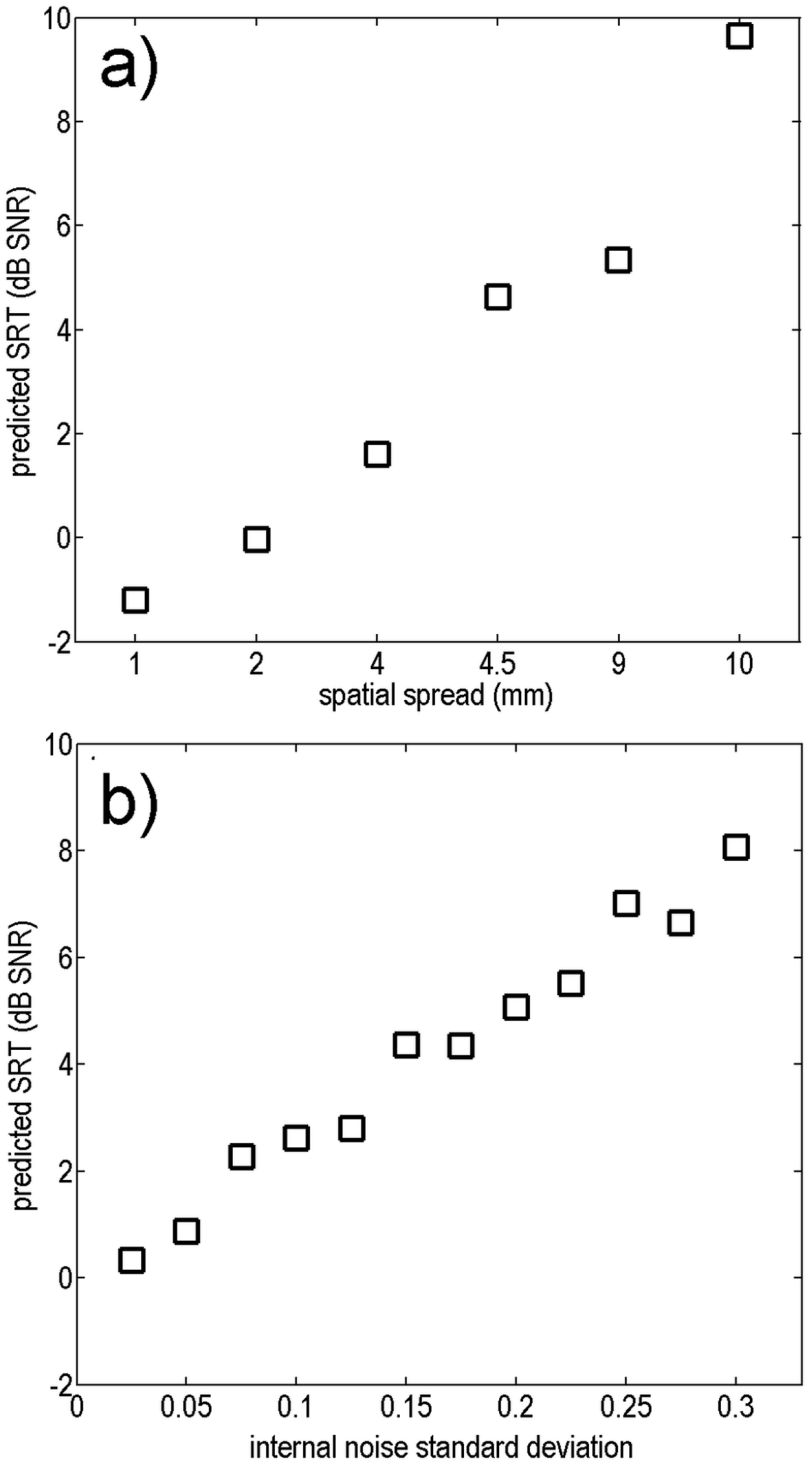
Prediction of SRTs with the speech intelligibility model (a) as a function of electrical field spatial spread with constant internal noise standard deviation σ_int_ = 0.19 and (b) as a function of σ_int_ with constant electrical field spatial spread (λ = 9 mm).

In Eq (1) Dur(mHL) is the duration of moderate hearing loss in years, Dur(sHL) is the duration of severe hearing loss in years, Δs is a factor that depends on the usage of hearing aids during the phase of severe hearing loss prior to implantation (-0.83%/y for no, -0.64%/y for one, and -0.45%/y for two hearing aids). These terms were taken from [16], who inferred these by investigating data of 2251 CI patients. Duration of moderate hearing loss is defined as the difference in years between first self-reported notice of hearing impairment to inability to use the telephone with the impaired ear. Duration of severe hearing loss is defined as the difference in years between inability to use the telephone to implantation date. B1 and B2 (both in %) are taken from [15], who investigated the same pool of CI patients. B1 and B2 reduce or increase the AP based on the patient’s age at implantation (B1, see [15] Fig 4) and etiology (B2, see [15] Fig 6).

The TRT-test result was used to quantify the (non-audiological) cognitive performance of the participant. Three different ways were realized to determine the individual σ_int_: (1) using the TRT-test result only, (2) letting TRT-test and anamnesis data contribute with equal weights and (3) using the anamnesis data only. Pilot testing with the model showed that a reasonable range of internal (multiplicative) noise standard deviations is between σ_int_ = 0.15 and σ_int_ = 0.25 (σ_int_ is a scalar without a unit). Therefore, the ranges of individual factors were then linearly mapped onto this range. This means that the poorest performer was assigned the highest noise standard deviation of 0.25 and the best performer was assigned the lowest σ_int_ (0.15). Table 2 shows individual σ_int_ values for all participants derived either using the aforementioned three combinations of TRT-test result and patient anamnesis. A color code was chosen to visually highlight good (green), moderate (black), and poor (red) performance. Note that adjustment of the internal noise due to the results of either of those tests can only be a very coarse model of limiting human cognitive performance and is not intended to model the details of functional or dysfunctional cognitive processes in human listeners.

**Table 2:** TRT-test results, auditory performance (AP), σ_int_ values derived using three different combinations of these TRT and AP for individual CI users, and SRTs of individual CI users. Red values indicate poor, black values medium, and green high performance.

CI user ID	TRT-test result (%)	AP (%)	Internal noise σ_int_ derived from			SRT (dB SNR)
			TRT-test only	TRT-test and AP	AP only	
08	44.9	-17.6	0.205	0.211	0.216	2.7
12	62.7	-15.8	0.150	0.180	0.211	2.0
30	41.6	5.4	0.215	0.183	0.150	6.2
31	60.7	-5.8	0.156	0.169	0.182	0.8
32	30.4	0.7	0.250	0.207	0.164	6.2
41	38.4	-17.0	0.225	0.220	0.214	4.5
44	50.3	-2.6	0.188	0.181	0.173	4.0
46	49.8	2.6	0.190	0.174	0.158	2.9
48	54.5	-0.7	0.175	0.171	0.168	-0.1
50	49.5	-14.8	0.191	0.199	0.208	1.6
60	55.1	-29.5	0.174	0.212	0.250	-0.1
61	52.7	2.2	0.181	0.170	0.159	1.7
62	56.1	-5.0	0.170	0.175	0.180	4.4
63	38.9	-17.0	0.223	0.219	0.214	3.9
Average	49.0	-8.2	0.193	0.191	0.190	2.9

### Generalized linear model

A generalized linear model (GLM) was used to assess the predictive power of each of the three individually extracted parameters: average FWHM of the spatial spread, total auditory performance (AP), and TRT-test result. Statistical independence and a linear combination of the three normally distributed variables were assumed.

### Experiment 3: Different degrees of model individualization

Experiment 3 investigates the question if an individual incorporation of either the EFI data (assessing the electrical field spatial spread) or the internal noise (σ_int_ parametrized by the TRT-test data or the AP or both) into the physiological model of CI user’s speech intelligibility can improve the prediction SRTs. Therefore, a step-wise approach was taken using three sub-experiments:

Experiment 3a: Internal noise individualization using either only the AP, only data from TRT-test, or a combination of both AP and TRT-test with equal weights.
Experiment 3b: Electrical field spatial spread individualization only
Experiment 3c: Full individualization of electrical field spatial spread and internal noise with noise strength estimated from either only the AP, or only data from TRT-test, or data from both AP and TRT-test with equal weights.

## Results

Three experiments have been designed to assess the efficacy of the model to predict SRTs of CI users. Experiment 1 performs a systematic analysis of the different parameters of the physiologically-inspired CI model described in the methods section. Experiment 2 presents the individual factors measured in CI subjects which may either in isolation or combined (linearly) correlate with speech performance. Finally, experiment 3 incorporates the individual factors into the physiologically-inspired CI model and compares the model predictions with the actual speech performance measured in each CI user.

### Experiment 1: Systematic model evaluation

Fig 4a shows SRT predictions varying the electrical field spatial spread (in the form of the parameter λ) systematically and uniformly across all electrodes. An average σ_int_ = 0.19 was chosen for this model variation, as this value is also used as an average for the internal noise strength for model individualization in experiment 3. Predicted SRTs increase (i.e., speech-in-noise discrimination is poorer) systematically as the electric field spatial spread of the model widens.

Fig 4b shows SRT predictions varying the internal noise strength systematically. An average electrical field spatial spread function of λ = 9 mm was chosen also for this model variation. Predicted SRTs increase, as σ_int_ increases. Note that the test-retest reliability of the predicted SRTs was calculated to 0.4 dB, based on several repetitions of predicting the same SRT.

### Experiment 2: Linear models of measurement results from individual CI listeners

#### Correlations of raw measurement data

**Fig 5:**
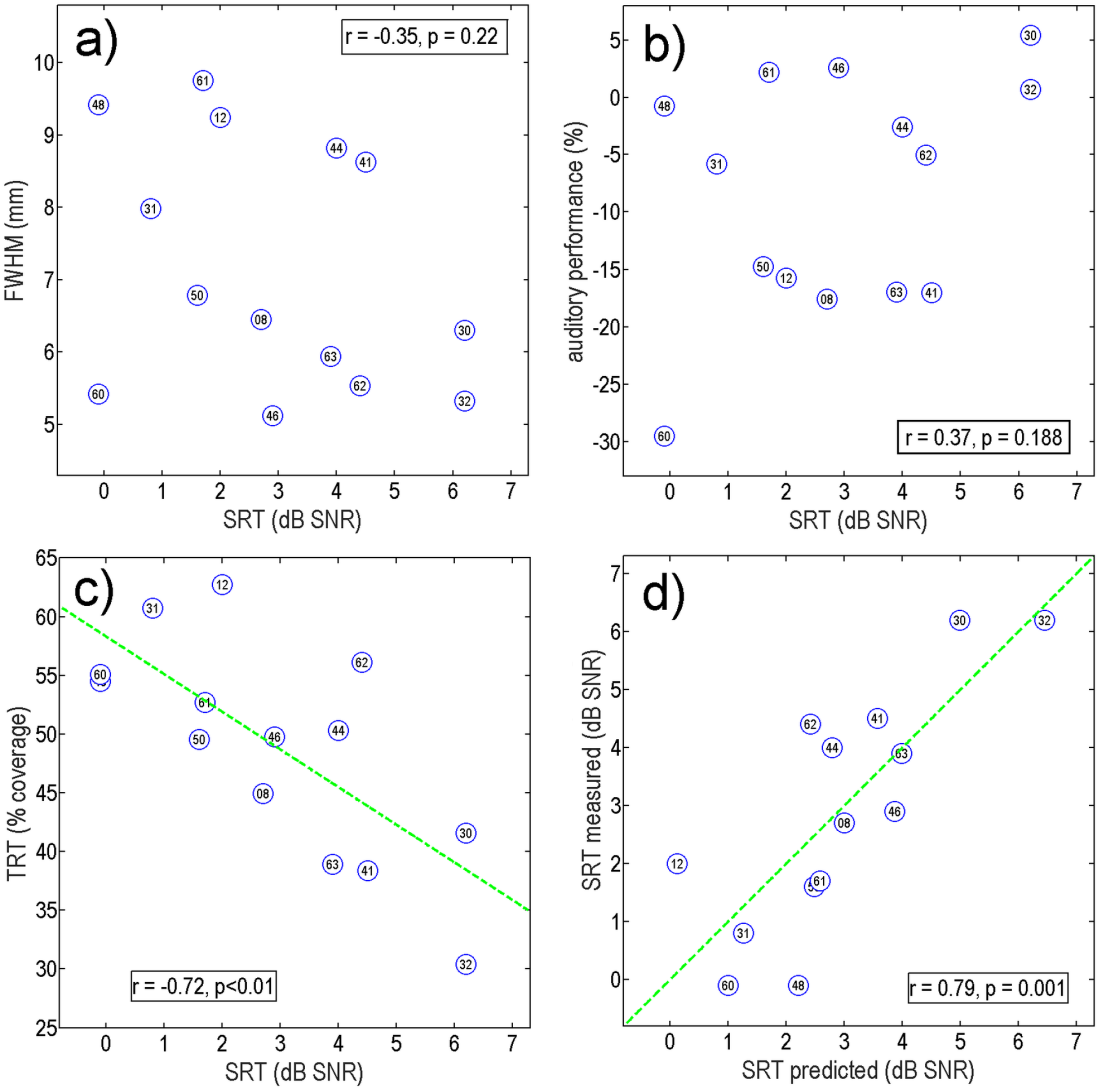
Correlation scatter plots of raw experimental data of all 14 CI users (with subject IDs) plotted versus measured SRTs. Panel (a) average FWHM of electrical field spatial spread, (b) “auditory performance” determined from anamnesis data using the phenomenological model of [16] and [15], (c) Text-reception-threshold data (% text coverage). Panel d) shows SRT-predictions of the generalized linear model (GLM).

Fig 5 shows scatter plots of average FWHM of the spatial spread (panel A), auditory performance (panel B), and TRT-test result (panel C) on the ordinate against individual SRT. Each participant is denoted using her/his ID number. The range of SRTs covered by the participants is between -0.1 dB SNR and 6.2 dB SNR, which corresponds to the range of SRTs covered in the systematic model evaluation (see Fig 4).

Neither the average electrical field spatial spread (Fig 5 panel a), nor the auditory performance alone (Fig 5 panel b) correlated strongly with the measured SRT using Pearson’s correlation coefficient (i.e., linearly). The trend of the (non-significant) correlation even showed the opposite sign than expected beforehand (wide spatial spread tended to be related to low SRTs and high auditory performance tended to be related to high SRTs). In contrast, the TRT-test result in the form of percentage of tolerated sentence coverage (Fig 5 panel c) correlated highly, r = -0.72 (p < 0.01) with measured SRT, indicating that participants, who could well combine fragments of words in a written sentence showed also better speech-in-noise performance and vice versa. The most probable linear regression line is plotted (green dashed) in those panels with significant correlations.

#### Predictions using a generalized linear model

A generalized linear model (GLM) was used to assess the predictive power of each of the three parameters: average FWHM of the spatial spread, auditory performance (AP), and TRT-test result. The fitted GLM can be described by Eq (2):

$$SRT_{pred}\left(dB\right)=11.62-0.0183\cdot FWHM\left(mm\right)+0.0644\cdot AP-0.1403\cdot TRT\left(\%\right)$$ (2)

The GLM-predicted SRTs as a function of the measured SRTs are shown in Fig 5d). The fitted GLM provided a significantly better prediction than the null hypothesis of a constant model (F = 5.57, p = 0.017). In line with the correlation analyses above, only the TRT-test result provided significant predictive value for the SRT (p = 0.015). The SRTs predicted by the fitted GLM showed a highly significant correlation coefficient with measured SRTs (r = 0.79, p = 0.001), explaining 62% of the total variance.

### Experiment 3: Model individualization

Three different degrees of individualization in the physiological model of CI user’s speech intelligibility were tested: One version that individualizes the electric field spatial spread only, one version that individualizes the internal noise only, and one that individualizes both factors combined. These three model versions were chosen to get a comprehensive picture about which factors are crucial in a nonlinear model mimicking speech-in-noise performance of CI listeners.

Table 3 shows Pearson’s correlation coefficient, the probability p that the null hypothesis of no correlation between measured and predicted SRTs needs to be rejected, RMS-error, and Bias between measured and predicted SRTs. In general, the model shows a negative bias of 2–3 dB with respect to the measured data, i.e., it underestimates the average performance of the listeners. There is only one significant correlation within the table of results: If the model’s internal noise is individualized to the TRT-test result only (taking an average spatial spread that is uniform across all electrodes), the highest correlation between measured and predicted SRTs is obtained. These SRT-predictions correlate highly significantly (p < 0.01) with SRT-measurements (r = 0.68). No significant correlations were found when individualizing both the internal noise and the electrical field spatial spread in combination, or when individualizing the electrical field spatial spread only.

**Table 3:** Evaluation of SRT-predictions by the physiologically inspired CI model outlined in experiments 3a-c) involving different forms of model individualization, which include contributions of the factors auditory performance (AP), text-reception-threshold (TRT) test, and internal noise standard deviation σ_int_.

		Correlation between model and measurements			
		Pearson’s corr. coeff.	p	RMS-error (dB)	Bias (dB)
internal noise standard deviation σ_int_ individualization	from AP	-0.35	0.22	3.1	-2.1
	from AP and TRT	0.32	0.26	2.9	-2.2
	from TRT	**0.68**	<0.01	2.9	-2.3
Electrical field spatial spread individualization only		-0.16	0.59	5.8	-2.8
Electrical field spatial spread and σ_int_ individualization	σ_int_ from AP	-0.18	0.54	6.2	-2.5
	σ_int_ from AP and TRT	-0.08	0.78	6.3	-2.7
	σ_int_ from TRT only	-0.04^1^	0.91	6.7	-3.1

**Fig 6:**
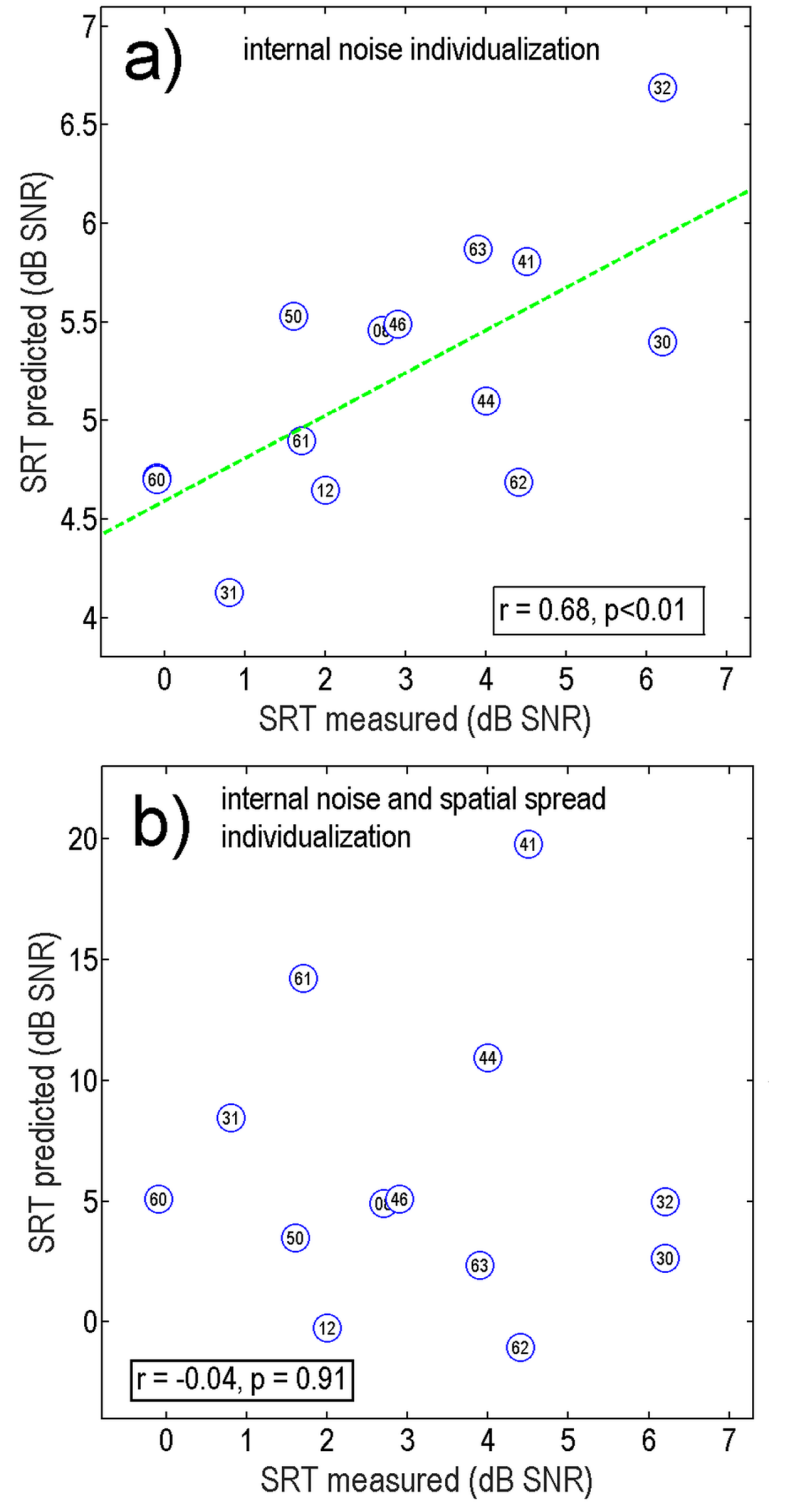
Correlation scatter plots of model-predicted and measured SRTs with individualization of (a) internal noise due to TRT-test results alone, and (b) with individualization of both electrical field spatial spread and internal noise. Note that the data point of participant 48 is hidden behind the data point of participant 60 in panel (a).

Fig 6 shows scatter plots (predicted vs measured SRTs) with a part-individualized model version (individualizing internal noise only from TRT-test result) in panel (a) and a full individualization (internal noise also from TRT-test) in panel (b).

When individualizing internal noise only, the high correlation (r = 0.68) to measured SRTs is clearly visible in panel a): predicted SRTs follow a diagonal direction with respect to measured SRTs. However, the model produces SRTs (around 4 to 7 dB SNR) that are more in line with listeners showing poorer SRTs and there remains a bias towards listeners with better SRTs. The highly significant correlation found with individualizing the model using internal noise only (panel a) is lost if additionally the electric field spatial spread is individualized (panel b). Note that the model predicted speech intelligibility scores below 50% for all SNRs tested for participant 048 in Fig 6 panel b. Therefore it was not possible to predict an SRT for this listener. The listener was thus excluded from the correlation coefficient calculation in this panel. The range of predicted SRTs is small (4 to 7 dB SNR) when individualizing internal noise alone and is sufficiently larger (-2 to 20 dB SNR) when individualizing both factors, which highlights the nonlinear behavior of the model with respect to these two factors.

## Discussion

This study systematically evaluated whether linear tools or a (nonlinear) state-of-the-art CI computer model can be used to predict individual speech performance of real CI users. The effect of individualizing different parameters of the front-end model (electrical spatial spread, cognitive noise) for predicting individual speech performance in a group of 14 CI users was investigated. In general, the results of non-individualized versions of the computational model show that the model predicts an improvement (decrease) of individual SRTs with narrower electrical field spatial spread and smaller internal noise standard deviation σ_int_ in agreement with the expectation. However, only an incorporation of σ_int_ estimated from the individual TRT-test result shows highly significant correlations to measured SRTs in CI subjects. The amount of correlation is of the same magnitude as the raw (linear) correlation between TRT-test and SRT. An additional incorporation of electrical field spatial spread renders this correlation insignificant.

### Effect of front end

The two factors electrical field spatial spread and σ_int_ within the model front end both showed considerable influence on predicted SRTs in the systematic evaluation (experiment 1). In line with predictions by [19] and [26] using the same model front end, a systematic decrease in electrical field spatial spread or in σ_int_ improves (decreases) SRTs. In the model, the wider electrical field spatial spread functions will cause wider modelled neural excitations, resulting in spectrally smeared IRs. With regard to spectral resolution, the same trend can also be found in vocoder studies, i.e., studies with NH subjects listening through an acoustical simulation of the CI user’s signal processing. Vocoder studies indicated that the number of independent frequency channels in CI users is effectively much lower than in NH listeners without vocoder processing [31], which limits speech-in-noise performance. The overlapping bandwidth of the vocoder channels spectrally smears the vocoder output and thus limits the spectral resolution. This affects speech recognition with higher speech scores for narrower bandwidths [32], improved SRTs for steeper vocoder filter slopes [33], and poorer speech scores going alongside reduced spectral ripple discrimination [10]. These vocoder studies (similar as in computer model studies) carry potentially less individual variability than studies with actual CI users due to the systematic control over the spectral resolution in the vocoder and a larger homogeneity across the NH subjects.

In actual CI users, however, the literature gives a less coherent picture about the effect of spectral resolution on speech perception. Psychophysical measures of spectral resolution [34], [5], evoked potentials [35], and spectral shape perception [36],[37] have been reported to correlate in varying degrees to speech perception. Highest correlations were found using those measures that assess spectral resolution across the whole cochlea, possibly because the stimuli used in these tests are closer to actual (broadband) speech stimuli.

### Individual predictions

Computer models of CI listeners currently work well for contrasting different preprocessing algorithms and different acoustic situations [38] with averaging over CI individuals. Also within one CI listener high correlations between predicted and measured SRTs were found by [38] using an envelope-correlation measure based on the electrodograms generated by the individual user’s CI. In contrast to the study of [38], the current study focused on correlations (between predicted and measured speech performance) across individuals in one specific (standard) acoustic situation that is widely used as a clinical test. Such correlations across individual CI users in one test are very rarely reported. An exception is the study of Stadler and Leijon [23]. In their study, a simple model as well as a physiologically detailed model of signal processing in CI users was individually adjusted due to results of a subjective spectral discrimination task. They found that both models could account for a large proportion of the speech-in-noise performance variance measured in CI users with a standard speech test. However, the measure that [23] used to assess spectral resolution uses wide-band signals, which makes this spectral resolution task closer to a speech-in-noise task (providing potentially a higher predictive power from the raw data) than the measure of spectral resolution used in the current study. In the current study, the individualization of spatial spread is based on intra-scalar voltage distribution measures (EFIs) that are electrode-specific and completely objective, i.e., they can be measured without interaction by the CI user. The hypothesis is that wider voltage distributions in the scala tympani should lead to increased spatial spread and in turn to poorer speech intelligibility (higher predicted SRTs) both in the CI user and in the model. However, both the raw data and simulation data with individualized spatial spread by using measured EFIs on each electrode and CI user in the model were not found to correlate directly to the SRT or to reduce the RMS error in predicting the SRT in the physiologically-inspired computer model (2.9 dB for the non-individualized spatial spread and 6.3 dB for the individualized spatial spread, see Table 3). Even a decrease of correlation coefficient is found when spatial spread is individualized in addition to the internal noise. This, and the additional absence of correlation to SRTs using the electrode-averaged electric field spatial spread widths indicates that this peripheral factor (as measured in the current study) is not predictive for individual SRTs.

This result should be interpreted with caution, because it does not prove that human neural resolution has a negligible effect on speech-in-noise performance of CI users. It could also mean that the normalized electrical potential distributions across CI electrodes include variations that may not correspond to human neural resolution and are thus inadequate as a measure for these. Since model results in experiment 1 and other studies such as [23] have shown that the human neural resolution is an important factor for speech-in-noise predictions of actual CI users, it is worthwhile to pursue this research further. EFI, as measured in the present study, however, can be excluded as a technique yielding predictive value for SRT-prediction.

The internal noise standard deviation inferred from the TRT-test result showed a high predictive value with correlation coefficients ranging between r = -0.72 (raw TRT-test result correlated with SRT) and r = 0.68 (with the part-individualized model). This is in line with the data reported in [17], who found significant correlation between TRT and SRT in a much larger sample size of 90 CI users. Their correlation coefficient was substantially lower (r = -0.27) and it is currently unclear what the reason for the difference is. One difference is that the study [17] recruited participants with CIs from three different manufacturers and different signal processing strategies across and within one manufacturer, whereas the current study controlled for these variables. The fact that the model simulation with TRT-individualized internal noise led to a similar correlation as with the raw TRT data is not surprising, because in this model version the variance of the internal noise is artificially forced to correlate with the TRT results. However, the relatively high correlation coefficient suggests that the internal noise individualization is a meaningful way of representing some of the more central factors in the model.

### Limitations of the current study and other factors

This study focuses on the assessment of several, but not all individual factors that may contribute to individual speech-in-noise performance. One important other factor not implemented in the computer model so far is the involvement of the status of the afferent spiral ganglion cells. The EFI measure can roughly correspond to neural excitation of the spiral ganglion cells only if a homogenous distribution of functional AN cells is assumed and if the distance from electrode to the nervous tissue is constant along the electrode array. However, dendrites of AN cells may have retracted, AN cell density locally or totally decreased, or even dead regions of completely missing AN cells in the cochlea [39] may occur. Better diagnostics are needed in order to include this factor in an individualized CI model, because currently there is no reliable test to estimate the status of the afferent spiral ganglion cells non-invasively in CI listeners. A constant distance of the electrode to that part of the nervous tissue where action potentials are generated is a further hypothesis that may be reasonable, at least in the first turn of the cochlear spiral, due to the circular placement of the electrode array. Further factors that may play a role are different individual TCL and MCL values and different loudness-growth functions.

The internal noise, as it is applied in the present study, can only be a very coarse model of some of the cognitive processes that are involved in speech perception of actual CI users. From a signal processing point of view the internal noise is merely a distortion of the input signal to the central stage (the FADE speech recognizer) that remains unchanged in all model versions. To mimic more realistically differences in human cognitive processes, also variations in the back-end would be needed, such as smearing the state-transition probabilities of the trained HMM or randomly deleting some HMM states. This was out of the scope of the present study and even when doing so it would be hard to prove that such artificial modifications of the backend provide a good model for variations in cognitive processes in actual human listeners. Currently it is still unknown how to exactly model human cognitive speech processing and this paper has not improved our understanding of this problem.

Future enhancements of the model could include spread of excitation measures using ECAPs instead of EFI measures, because ECAPs may be a better measure of human neural resolution. However, since ECAP spread of excitation measures are produced by auditory nerve responses, they are subject to a “double-application” of the spatial spread function from the electrode to the auditory nerve. A deconvolution as proposed by [40] would be suitable to implement these measures in the CI model. Additional possibilities to improve the modeling of individualized measures of spectral spread include combinations of psychophysics and imaging data [41]. To refine the modeling of the individual electrode-nerve interface, more detailed 3-dimensional models based on computer tomography data might be helpful (cf., [28]) to use within the frontend of the model.

## Conclusions

This study systematically evaluated a nonlinear model of CI user’s speech-in-noise performance with respect to the model-inherent factors electric field spatial spread and internal noise. Furthermore, the hypothesis was tested if an individual assessment of these factors with incorporation into the model can result into an improvement of individual SRT prediction. The predictions were compared to predictions with linear standard tools. The following conclusions can be drawn:

1. Predicted SRTs decrease (improve) with narrower electric field spatial spread, and with smaller internal noise standard deviation.
2. Only an incorporation of internal noise standard deviation estimated from the individual TRT-test result shows highly significant correlations to measured SRTs. The amount of correlation is of the same magnitude as the linear correlation between TRT-test result and SRT. An additional incorporation of electrical field spatial spread, as measured using normalized data, renders this correlation insignificant.

This may suggest that spatial spread estimates from EFI data are not sufficient to capture individual differences in neural spectral resolution and hence differences in speech-in-noise performance. As the TRT-test has shown high predictive value in this study with a highly variable group of participants in terms of age and etiologies, the TRT test is recommended as an important factor for individual speech-in-noise performance. This factor can also be measured pre-surgically with the purpose of predicting SRTs post-surgically. This study shows that it is difficult to incorporate other factors into the individual prediction, at least with the simplifying assumptions that have been taken in the current study.

## Supporting information

S1 Table Data set containing additional patient data, FWHMs of spatial spread, SRTs of systematic model evaluation, correlations of raw measurement data, SRTs and correlations of measured and modelled SRTs with all tested model versions. (XLSX)

## Abbreviations

ACE: advanced combinational encoder
AN: auditory nerve
AP: auditory performance
CI: cochlear implant
dB: deciBel
DTW: dynamic time warping
ECAPs: electric compound action potentials
EFI: electrical field imaging
FADE: framework for auditory discrimination experiments
FWHM: full width half maximum
GLM: generalized linear model
IR: internal representation
NH: normal-hearing
RMS: root-mean-square
σ_int_: internal noise standard deviation
SNR: signal-to-noise ratio
SPL: sound pressure level
SRT: speech reception threshold
TRT: text reception threshold
